# Summary of Observations upon the Effect of the Galvanic Current upon the Auditory Nerve

**Published:** 1873-08

**Authors:** Clarence J. Blake

**Affiliations:** Boston


*Summary of Observations upon the Effect of the Galvanic Current upon the Auditory Nerve.* By CLARENCE J. BLAKE, Boston.

Since the publication of the experiments of Brenner, and the interest excited by his observations on the reaction of the auditory nerve, under the influence of the galvanic current, the application of electricity to the diagnosis and treatment of affections of the ear has become much more general, and not only so far as concerns the condition of the auditory nerve, but also in the treatment of diseases of the middle ear, as is evidenced in recent German and English publications.

In his Beleuchtungsbilder des Trommelfells, Prof. Politzer says: "With the advance which is daily being made in the diagnosis and treatment of diseases of the ear, the domain of nervous deafness is being rapidly encroached upon and diminished." To any one who has followed the progress of aural surgery during the past ten years, this remark is plainly true, but there still remains a class of aural affections of the nature of which the speculum and otoscope give us but little information, and for which the ordinary surgical measures afford but slight relief. To this class belong, not only those cases of deafness where the middle ear shows no symptom whatever of disease, but also those in which a disease of the tympanum may be accompanied by, or may mask an affection of the labyrinth, or of the auditory nerve; and it is in these cases that we have to seek some more subtle means of diagnosis and of treatment.

The conditions under which the galvanic current may be employed by the aurist with advantage, as a means of assistance in diagnosis, may be superficially classified as those of—

Chronic catarrhal inflammation of the middle ear of long standing, with considerable deafness and diminution or absence of the perception of sound conveyed through the bones of the head.

Chronic catarrhal inflammation of the middle ear of shorter duration, accompanied by tinnitus aurium and diminution of hearing.

Cases of deafness occurring during pregnancy, in child-bed, at puberty, or referred to mental disturbance, mental or bodily exhaustion, or accident, as the cause, in which the middle ear shows no signs of disease whatever, or not sufficient to account for the degree of deafness which may pertain in such cases.

Deaf-mutism, congenital or acquired.

The principle laid down by Prof. Brenner, to whom is due the credit of establishing a formula of reaction of the auditory nerve, under the action of the galvanic current, is, that "the auditory nerve in health reacts to the galvanic current in a regular manner by distinct sounds at and during the closure of the negative, and at the opening of the positive pole; that these sounds may be whistling, ringing, hissing, singing, humming, and rumbling; that they vary in different individuals, and are modified by the strength and duration of the current; that the most frequent is whistling, which is clear, agreeable and musical, and that any departure from these reactions indicates disease."

The statements of Brenner were at first denied by some observers, but were also and more recently largely confirmed by others, so that the formula established by him may be taken, not as a matter of conjecture, but as a positive contribution to our knowledge of the condition of the auditory nerve. A means is thus given of answering the question in any given case, as to whether the auditory nerve is or is not in a normal condition, and with this a point of decided value both as concerns prognosis and treatment. On the other hand, however, it is not always possible to establish a formula of reaction of the auditory nerve; indeed in many cases no formula whatever can be obtained, although the necessary conditions, with regard to a successful application of the current, are fulfilled. In such cases, the practical value of the discovery of Brenner is much diminished, and it becomes necessary to seek some other demonstration of the effect which the galvanic current may have upon the auditory nerve, and this may be accomplished by testing the effect which the current has upon its perceptive power.

The cathode being applied in front of the ear to be tested, and the anode held in the hand of the opposite side, and the current gradually increased, I have found the perception for high musical tones to increase from three to five thousand vibrations in the second above the highest limit of hearing before the application of the current. This increase in perception continued during the passage of the current, and for a short time after the opening of the cathode, but was speedily diminished by a reversal of the current and application of the anode. Ritter has observed, that on passing a direct continuous current through his own ears, the sound of a tuning fork, G, gradually increases in pitch with the increase in intensity of the current.

The cathode being applied in front of the ear, as in the previous experiment, we find that the passage of the galvanic current increases not only the limit of perception of musical tones, but also the intensity of perception, the degree of increase in intensity of perception being a measure of the degree in which the auditory nerve responds to the stimulus.

In establishing this observation, experiments were first made in cases of normal hearing, and later in those of diminished hearing, resulting from disease of the middle ear, or without visible cause. In all cases a current from a Stoehrer battery without the rheostate was employed, and the electrodes applied, the cathode in front of the tragus, and the anode to the hand of the opposite side. A tuning fork set in vibration by means of a spring hammer giving a stroke of uniform intensity, was first held opposite the ear to be tested, and the length of time during which the tone was heard noted; the same test was repeated under the stimulus of the galvanic current, and the increase in the duration of the perception also noted. In the healthy ear the increase is from one-twelfth to one-sixth of the normal duration of perception, on the average, and this proportion diminishes in accordance with the diminished reactive power of the auditory nerve.

As an illustration of this manner of employing the continued current, cases of chronic catarrhal inflammation of the middle ear, forming about 27 per cent. of all aural affections occurring in this climate, may be instanced. In the ordinary course of the disease there occurs first a diminution of patency of the Eustachian tube, rarefaction of the air contained within the middle ear, and pressure of the membrana tympani inwards, the thickening of the mucous membrane lining the tympanum, and forming the inner coat of the membrana tympani, tending to retain the latter structure in its abnormal position, thus producing an abnormal degree of intra-labyrinthine pressure, and preventing also the proper transmission of sonorous vibrations from without inwards to the labyrinth. As this condition continues and increases, there is gradual increasing diminution of hearing on account of the changes in the middle ear, and secondarily, a diminution of perceptive power of the auditory nerve, as shown by tests with the watch and tuning-fork, pressed upon the temple and held between the teeth. The use of the galvanic current in such cases, will serve to determine the degree to which the auditory nerve has become affected, and also the power of perception under stimulation remaining to it, the result materially influencing not only the prognosis but the treatment.

In those cases of chronic catarrhal inflammation of the middle ear in which the perceptive power is but slightly diminished, and where tinnitus aurium is a prominent symptom, the result of the use of the galvanic current is even more satisfactory. As a result of the progressive changes within the middle ear, there is, with the pressure of the membrana tympani and ossicula inward, evidence of the effect of this pressure and the accompanying irritation in the auditory nerve in the occurrence of subjective noises, noticed at first occasionally, and finally becoming constant, and increasing with the progress of the disease in the middle ear. In many of these cases it will be impossible to obtain a positive reaction according to the formula of Brenner, but we may be guided in the application of the current by the tests described, and in addition by the effect which the current has upon the tinnitus aurium. Its first application will of course be purely experimental.

The hearing having been carefully tested with regard to the hearing distance by a test watch, the perception of high musical tones, by means of Konig's rods, and the duration of perception of the tuning fork, and the patient's attention drawn to the degree of the tinnitus aurium, the cathode may be first applied in front of the ear, and then the anode of equal strength and for the same length of time, the hearing being again carefully tested, during and after the passage of either current.

It will be found as a rule that the current which diminishes the tinnitus aurium increases the hearing, and that the current which increases the tinnitus diminishes the hearing. In cases where the tinnitus is diminished without increase of the hearing, this effect is usually produced by the use of the anode, the cathode increasing the tinnitus, without a corresponding increase of hearing. When the hearing is increased, together with an increase of the tinnitus, this result is usually obtained with the use of the cathode.

In the use of the galvanic current in the treatment of these cases, we should be guided by the result of the experimental application; the current to be employed being the one which increases the hearing and diminishes the tinnitus, or, in default of this double effect, the current which increases the hearing, even at the expense of increasing the tinnitus, experience showing that the tinnitus, though temporarily increased, gradually subsides and diminishes with the improvement of hearing at subsequent applications of electricity. The following case is an illustration of the latter observation: The patient was a man in robust health, twenty-one years of age; the hearing had been gradually but noticeably decreasing for more than three years, and during that time there had been a constant rushing sound in both ears; this sound did not vary materially with change of climate, weather, or condition. The membrana tympani of both ears was concave, opaque and of a dull gray color. The use of the air douche and catheter did not improve the hearing, which was $\frac{1\frac{1}{2}}{72}$ in the right ear and $\frac{\frac{3}{4}}{72}$ in the left ear, as tested by the watch. The perception through the bones of the head, for the watch, was better in the left ear, and for the tuning-fork, better in the right ear, but diminished in both. The current from a Stoehrer battery was applied, without obtaining formula of reaction; the use of the anode neither diminished the tinnitus aurium nor improved the hearing; the use of the cathode increased the tinnitus aurium, and also improved the hearing. The use of the cathode was therefore continued at intervals of two days, and after the third application, the hearing tested by the watch had increased to $\frac{4\frac{1}{2}}{72}$ in the right ear and $\frac{3}{72}$ in the left ear, accompanied by a sensible diminution of the tinnitus aurium. In the following case also no positive formula of reaction could be obtained: A woman, thirty-five years of age, who had never been robust, and who had performed much mental labor, and who was five months pregnant at the time of treatment, had, in addition to the diminution of hearing consequent on a slight catarrhal inflammation of the middle ear, a continuous rushing sound in both ears, varied occasionally by rhythmical beating and a loud ringing. The use of the cathode increased the tinnitus, but did not increase the hearing; the use of the anode, however, increased the hearing and diminished the tinnitus aurium, which did not increase to its former degree until three or four days had elapsed. By repeated use of the anode, during a period of six weeks, the tinnitus disappeared entirely and the hearing materially improved. In the case of a woman fifty years of age, the hearing having gradually diminished during a period of five years, and more especially since a severe mental shock, until it was but $\frac{2}{72}$ in the right ear, and $\frac{2\frac{1}{2}}{72}$ in the left ear, the galvanic current gave the normal formula of Brenner. It was found, however, that the use of the cathode increased the hearing distance, and also the perception for musical tones, and diminished the tinnitus aurium, which had been constant and annoying. After the fifth application of the cathode, the hearing had increased to $\frac{6}{72}$ in the right ear, and $\frac{7}{72}$ in the left ear. In this case the membrana tympani was transparent, and normal in position, the Eustachian tubes perfectly free, and the hearing was not increased by the use of the air douche.

As an instance of the action of a stimulant current upon the auditory nerve in a patient, in whom the hearing was not materially improved, the following case may be mentioned:—

A man thirty-two years of age, first noticed diminution of hearing and a rushing sound in both ears, ten years before the date of his application for treatment. His general health had been good, with exception of occasional attacks of malarial fever, for relief from which he had used quinine in large quantity. The hearing had so far diminished, that the watch was not heard when pressed upon the auricle, and the tuning-fork held between the teeth, was heard only in the left ear. In the right ear, however, a tone of 50,000 v. s. was distinctly heard, and in the left ear a tone of 45,000 v. s. The membrana tympani of both ears was transparent and apparently normal, except that it was quite concave. The hearing was not improved by the use of either Politzer's air douche, or the catheter. No formula of reaction could be obtained, but the use of the cathode increased the perception for high musical tones to 60,000 v. s., and for duration of hearing of the tuning-fork, from twenty seconds to thirty-five seconds.*

This increase in the perception for musical tones persisted but a short time after the application of the current, the duration of perception of the tuning-fork, however, continued to increase, but never reached the normal standard, and the improvement in the general hearing was, on the whole, so very slight, that the use of the current was finally abandoned. This case presented several points of interest. So far as could be determined, the middle ear was in a healthy condition, with exception of such changes as were evidenced by the concavity of the membrana tympani. Assuming the auditory nerve to be in a normal state, we should expect an increase in the limit of perception for high musical tones, as was the case, on account of the increased tension of the membrana tympani; the same condition would tend to diminish the hearing for lower tones, as was the case in the test with the tuning-fork, and in the hearing for the voice. The use of the cathode increased the hearing for the voice, and for the watch slightly, and decidedly increased the perception for high musical tones, and also the duration of the perception of the tuning-fork; and an excision of a portion of the membrana tympani, which operation was subsequently performed, had no appreciable effect upon the hearing whatever.†

* The tuning-fork, A 562 v. s., being struck by the spring-hammer with a force equivalent to one pound falling one inch, the normal duration of hearing is from 55 to 60 seconds.

† In the majority of cases in which this latter operation is performed, the perception for high musical tones is immediately increased; in one case in which

For practical purposes, in the ordinary routine of aural practice, the galvanic current may be employed in the simplest manner, without the intervention of the rheostate, and by applying one electrode in front of the ear and the other to the hand of the opposite side; at least my own experience has led me to content myself with this method of application.

In many of the cases belonging to the classes cited, in which it might be deemed advisable to employ the galvanic current as a means of diagnosis, it may prove impossible to obtain any of the definite formulæ of Brenner. It is in such cases that the tests and reactions above described may be of service.—*Archives Scientific and Prac. Med.*

